# Quantum Confinement Suppressing Electronic Heat Flow below the Wiedemann–Franz Law

**DOI:** 10.1021/acs.nanolett.1c03437

**Published:** 2022-01-14

**Authors:** Danial Majidi, Martin Josefsson, Mukesh Kumar, Martin Leijnse, Lars Samuelson, Hervé Courtois, Clemens B. Winkelmann, Ville F. Maisi

**Affiliations:** † Université Grenoble Alpes, CNRS, Grenoble INP, Institut Néel, 25 rue des Martyrs, 38042 Grenoble, France; ‡ NanoLund and Solid State Physics, Lund University, Box 118, 22100 Lund, Sweden

**Keywords:** heat transport, quantum dot junction, scattering theory, Wiedemann−Franz law

## Abstract

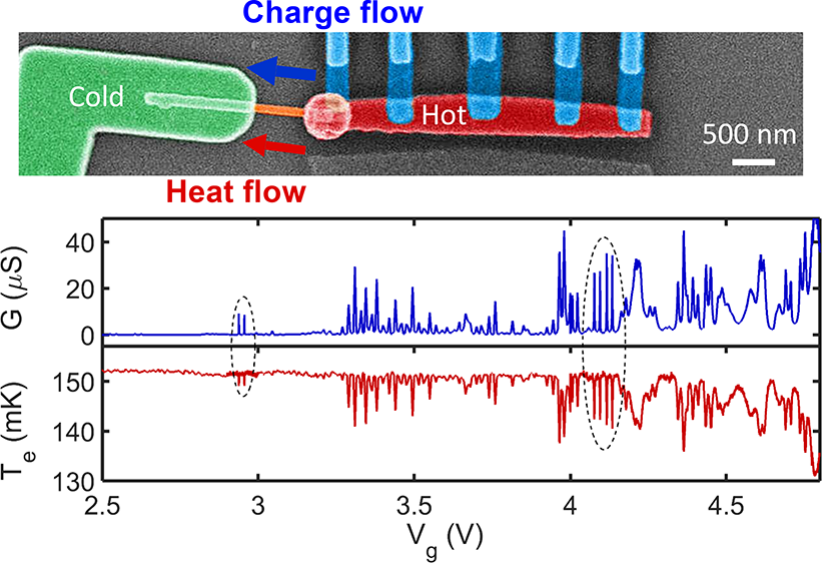

The Wiedemann–Franz law states that the charge conductance and the electronic contribution to the heat conductance are proportional. This sets stringent constraints on efficiency bounds for thermoelectric applications, which seek a large charge conduction in response to a small heat flow. We present experiments based on a quantum dot formed inside a semiconducting InAs nanowire transistor, in which the heat conduction can be tuned significantly below the Wiedemann–Franz prediction. Comparison with scattering theory shows that this is caused by quantum confinement and the resulting energy-selective transport properties of the quantum dot. Our results open up perspectives for tailoring independently the heat and electrical conduction properties in semiconductor nanostructures.

In conductors, a higher electrical conductance *G* is generally associated with a correspondingly higher heat conductance κ. The Wiedemann–Franz (WF) law indeed stipulates that at a given temperature *T*, the ratio defined as *L* = κ/*GT* is constant and equal to the Lorenz number *L*_0_ = (π^2^/3)(*k*_B_/*e*)^2^. The connection of the two quantities arises from the fact that the particles responsible for the transport of charge and heat, respectively, and the relevant scattering mechanisms are the same. Experimentally, the WF law has been verified to hold down to the scale of single-atom and molecule contacts.^1,2^ When phonon contributions^3^ can be neglected, deviations indicate departures from Fermi liquid physics^4^ such as found in superconductors,^5^ correlated electron systems,^6,7^ Majorana modes,^8^ or viscous electron flow.^9^ In quantum nanodevices, Coulomb interactions and charge quantization were also shown to lead to departures from the WF law.^10−13^

In semiconducting materials, the WF law is usually well obeyed for the electronic contribution to heat conductance, including semiconducting nanostructures displaying transport in the quantum Hall state.^14,15^ This property imposes severe limitations for instance in thermoelectrics, for which it is desirable to maximize the charge flow while minimizing that of heat. The most common figure of merit for thermoelectric conversion, *ZT* = *GS*^2^*T*/κ, where *S* is the Seebeck coefficient, is indeed directly proportional to *L*^–1^. Nevertheless, semiconducting nanostructures can display adjustable and strongly energy-selective transport processes, which could also lead to breaking the WF law, even in the absence of interaction effects.^10,16,17^ This can be provided for instance by the quantization of the energy levels in a single-quantum-dot junction, allowing for an adjustable narrow transmission window in energy. Although theory has predicted a vanishing *L*/*L*_0_ for weakly tunnel-coupled quantum dots at low temperature,^18−23^ it was experimentally shown that higher-order effects restore a significant electronic heat leakage.^24^ The validity of the WF law in a single-quantum-dot device has however not yet been quantitatively investigated because of the difficulty in measuring the extremely small heat currents.

In this work, we investigate heat flow in a quantum dot formed in an InAs nanowire grown by chemical beam epitaxy.^25^ Such nanowires have been widely studied for their promising thermoelectric properties.^26−30^ It was recognized that the formation of quantum-dot-like states in nanowires can lead to a large enhancement of the thermopower, well beyond expectations from 1D models.^26^ Such quantum dots can be produced either by inserting controlled InP tunnel barriers or simply by the inherent electrostatical nonuniformities at a low carrier density. They recently allowed experimentally testing the Curzon–Ahlborn limit of thermoelectric conversion efficiency at maximum power.^31^ Although entering directly in the thermoelectric efficiencies, the electronic heat conductance of such devices is in general not measured independently. Because at temperatures above a few degrees Kelvin, the thermal transport properties of InAs nanowires are known to be strongly dominated by phonons,^32^ the *electronic* heat conductance of InAs can only be experimentally probed at milliKelvin temperatures.

The experimental device is an InAs nanowire of 70 nm diameter, back-gated from the degenerately doped silicon substrate at a potential *V*_g_ and electrically connected on one side to a large gold contact named *drain* from hereon (Figure 1a). The contact resistance to such nanowires is typically on the order of a few 100 Ω at most,^33,34^ that is, much less than the device resistances that we consider in this work. The nanowire conductance d*I*/d*V*_NW_ is measured using a voltage division scheme as pictured in Figure 1a, involving a 10 MΩ bias resistor. The other side (the *source*) consists of a few-micrometer-long normal metallic island, connected by five superconducting aluminum leads. The leftmost of these in Figure 1a is in direct ohmic contact with the source island. This allows measuring directly the nanowire linear charge conductance *G*(*V*_g_), as shown in Figure 1c. In agreement with previous reports on similar structures,^26^ the nanowire conduction is pinched off below *V*_g_ ≈ 3 V. Near the pinch off, the conductance displays sharp resonances, which indicate that the nanowire conduction bottleneck at vanishing charge carrier densities will be provided by a quantum dot forming in the part of the nanowire that is not below the metallic contacts (Figure 1c). Although “unintentional” (in contrast with epitaxially engineered quantum dots^35,36^), these quantum dots display a well-defined level quantization *δε*, tunnel coupling strengths γ_s,d_, and charging energies *E*_c_ all three significantly larger than *k*_B_*T*. Here, *k*_B_ is the Boltzmann constant, and *T* is the experimental working temperature, which is set to *T*_b_ = 100 mK at equilibrium. Details of the charge conductance properties, which we extract from full d*I*/d*V*_NW_(*V*_NW_, *V*_g_) differential conductance maps, are found in the Supporting Information.

**Figure 1:**
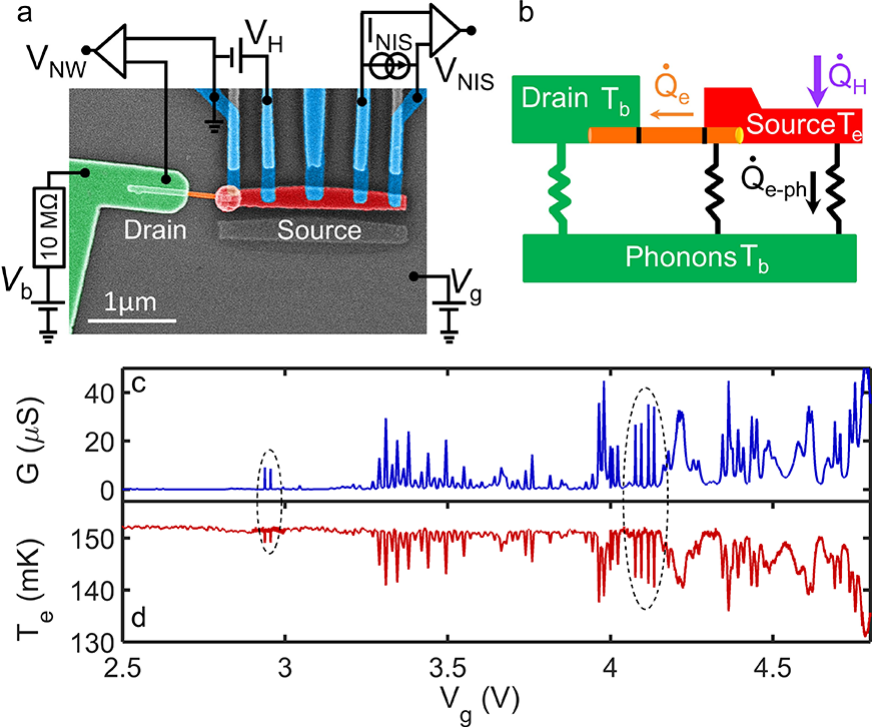
Heat transport experiment through an InAs nanowire device. (a) False-colored scanning electron micrograph of the device. The drain electrode, the source island, and the nanowire are colored in green, red, and orange, respectively. Five superconducting aluminum leads (light blue) are connected to the source island for heating the source side and measuring its electronic temperature. Thermometry is performed by measuring the voltage *V*_NIS_ at a fixed floating current bias *I*_NIS_. (b) Heat balance diagram, which includes the applied power to the source island, *Q̇*_H_; the heat escaping due to electron–phonon coupling, *Q̇*_e-ph_; and the electronic heat flow along the nanowire, *Q̇*_e_. (c) Electrical conductance at thermal equilibrium and (d) temperature response *T*_e_ of the source island with the heating power of *Q̇*_H_ = 16 fW as a function of the back gate voltage *V*_g_. The dashed ellipses highlight resonances that will be studied in more detail. All measurements are taken at a bath temperature *T*_b_ = 100 mK.

The other four aluminum leads to the source are in contact via tunnel barriers. Such superconductor–insulator–normal metal (NIS) junctions are well-known to provide excellent electron heaters and thermometers in low-temperature experiments.^37^ Because at mK temperatures both the electron–phonon coupling in metals and the heat conductance of superconductors are very low, the source island electrons are thermally well insulated, such that the heat flow through the nanowire significantly contributes to the source island’s heat balance. This is seen in Figure 1d, in which a constant heating power *Q̇*_H_ = 16 fW is provided to the source island via a voltage *V*_H_ applied on one tunnel lead. As the gate potential is swept, the variations of the source island electron temperature *T*_e_ are strikingly anticorrelated to variations of *G*. The heat balance of our device is schematized in Figure 1b. Because the source island is overheated with respect to its environment, the gradual opening of electronic conduction channels in the InAs nanowire leads to increased heat flow out of the source island and thus a lowering of *T*_e_.

In the remainder of this work, we investigate quantitatively the nanowire heat conductance properties and compare them to the predictions of both the WF law and the Landauer–Büttiker scattering theory.^38^ To this end, it is very insightful to go beyond linear response in Δ*T* = *T*_e_ – *T*_b_, and we thus measure at every gate voltage the full relation *Q̇*_H_(*T*_e_,*V*_g_) between the Joule power *Q̇*_H_ applied to the source and its internal equilibrium electronic temperature *T*_e_. Details of the determination of *Q̇*_H_ are described in the Supporting Information.

An important issue in the determination of electronic heat flow is the proper identification of the parasitic heat escape via other channels, such as electron–phonon coupling.^37^ Unless the latter can be neglected,^14^ the comparison to a reference, at which the electronic heat conductance is either assumed to be known,^12^ or negligible, is required. We define *Q̇*_H_(*T*_e_,0) measured deep in the insulating regime as an experimental reference which contains all heat escape channels out of the source island other than mediated by the nanowire charge carriers. We stress that this choice does not rely on any thermal model, and we furthermore consider the gate-dependent part of the heat balance, defined as *Q̇*(*T*_e_,*V*_g_) = *Q̇*_H_(*T*_e_,*V*_g_)–*Q̇*_H_(*T*_e_,0). The magnitude and temperature dependence of *Q̇*_H_(*T*_e_,0) is in good agreement with estimates for the electron–phonon coupling in the metallic parts of the source (see Supporting Information). Surprisingly, we observe that *Q̇*(*T*_e_,*V*_g_) is slightly gate dependent even before the conducting state sets on. This is readily visible as a slightly negative slope of the *T*_e_(*V*_g_) baseline in Figure 1d. We thus conclude on a minute yet measurable and smoothly gate-dependent contribution to the source electron–phonon coupling from the part of the nanowire below the source, which calls for defining in addition a local reference, as discussed below.

The very first conduction resonance, visible in Figure 1c,d and Figure 2a,b at *V*_g_^0^ = 2.938 V, is ideally suited for a *local* background subtraction, revealing the electronic heat conductance *Q̇*_e_ through the nanowire on top of the smooth e-ph background contribution *Q̇*_e-ph_ of the source side. At gate voltages |Δ*V*_g_| ≥ 3 mV away from the conduction resonance at *V*_g_^0^, the heat flow *Q̇*(*T*_e_,*V*_g_) is constant, within noise, although the charge conductance *G* still varies. After taking the difference of the heat balance on and off resonance (Figure 2c), one is thus left with the quantity of interest, the *electronic* heat flow through the nanowire at resonance, *Q̇*_e_(*T*_e_,*V*_g_^0^) = *Q̇*(*T*_e_,*V*_g_^0^) – *Q̇*(*T*_e_,*V*_g_^0^ + *ΔV*_g_). We stress that this additional background subtraction does not rely on any modeling of the heat balance, such as electron–phonon coupling. As seen in Figure 2d and already visible in the inset of Figure 2c, *Q̇*_e_ at *V*_*g*_^0^ displays a strikingly linear dependence on Δ*T*. We see that the heat conductance κ_e_ = ∂*Q̇*_e_/∂*T*, that is the initial slope in Figure 2d, differs quantitatively from the WF prediction by a factor *L*/*L*_0_ ≈ 0.65 ± 0.1. Further, beyond linear response, the temperature dependence qualitatively deviates from the parabolic law expected from WF (dashed line).

**Figure 2:**
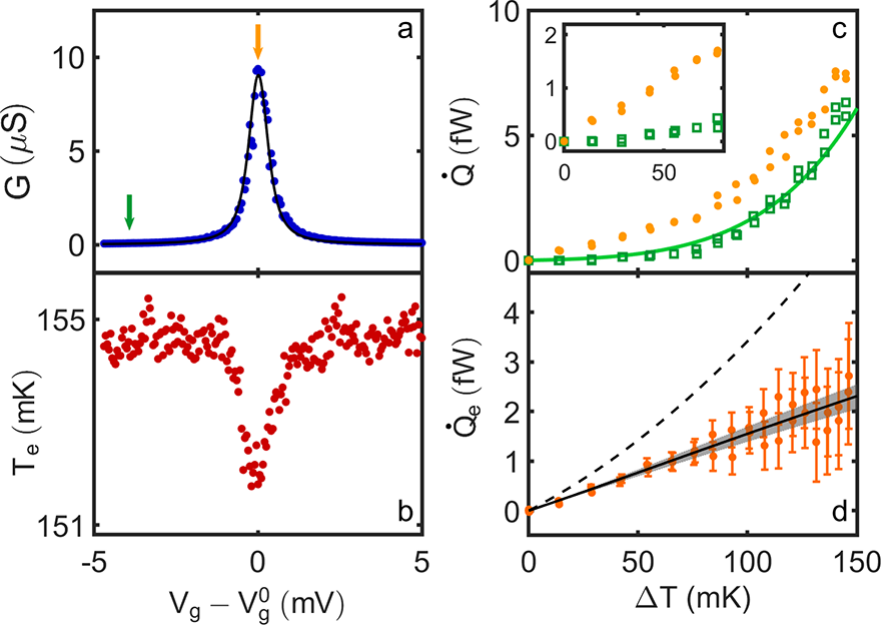
Heat transport near an isolated conductance resonance. (a) Linear charge conductance around *V*_g_^0^= 2.938 V at *T*_b_ = 100 mK. The black line is a fit using scattering theory. (b) Source temperature *T*_e_ as a function of *V*_g_, with a constant applied power *Q̇*_H_ = 16 fW, at *T*_b_ = 100 mK. (c) Full heat balance curve *Q̇*(*T*_e_,*V*_g_) on (orange bullets) and off (green squares) the transport resonance, as indicated by the arrows in (a). The green line presents a fit using *Q̇* = β(*T*_e_^6^–*T*_b_^6^) with β = 35 ± 5 pW/K^6^. The inset highlights the electronic contribution, dominating at the small temperature difference at the resonance. (d) The difference of the two data sets in c, displaying the purely electronic heat transport contribution *Q̇*_e_. The dashed and the full lines are the predictions from the WF law and scattering transport theory, respectively. The gray shaded area indicates the uncertainty of the scattering theory calculation, due to the determination of the gate coupling lever arm. The error bars account for the uncertainty in the experimental determination of *Q̇*_e_.

For a theoretical description beyond the WF law, we use a Landauer–Büttiker noninteracting model, with an energy-dependent transmission . We write the associated charge and heat currents, respectively as
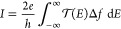
1and
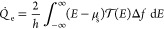
2with Δ*f* the difference in the source and drain energy distributions, and μ_s_ the source island chemical potential.^38,39^ The linear charge and heat conductances are then obtained as *G* = *∂I*/*∂V*_NW_ and κ_e_ = ∂*Q̇*_e_/∂(*ΔT*), respectively, with Δ*T* = *T*_e_ – *T*_b_. We model each resonance as a discrete energy level coupled to the source and drain reservoirs. We then deduce the transmission function  by fitting the calculated gate-dependent charge conductance *G*(*V*_g_) to the data. The accurate determination of  requires accurately estimating independently the tunnel couplings and the gate lever arm, as both affect similarly the resonance widths. This is described in detail in the Supporting Information. On a technical note, we stress that the above theoretical expression of κ_e_ assumes open-circuit conditions, that is, no net particle current. For all heat conductance experiments, the nanowire was biased in series with a 10 MΩ resistor at room temperature. Because we only consider data at gate voltages at which *G* is significantly larger than (10 MΩ)^−1^ = 0.1 μS, applying *V*_b_ = 0 is then equivalent to imposing open circuit conditions.

With the above analysis, the Landauer–Büttiker theoretical *Q̇*_e_(*T*_e_,*V*_g_) follows directly. As seen in Figure 2d (solid black line), the agreement with the experimental data is very good, with no adjustable parameters, reproducing the observed approximately linear dependence on Δ*T*. The gray shaded region accounts for the uncertainties in the determination of . The violation of the WF law observed here is therefore accurately described by a noninteracting scattering transport picture.

Intuitively, the deviation from WF at resonance can be understood as stemming from the energy selectivity of the device transmission , which is a peaked function of width γ = γ_s_ + γ_d_, with γ_s__,__d_/*ℏ* the tunneling rates between the dot and the source and drain leads, respectively. Only electrons bound at the Fermi level within an energy window of width γ can effectively tunnel, thereby suppressing contributions of the particles from the high-energy tails of the Fermi distribution. Together with a large Seebeck coefficient,^26,27^ this relative suppression of heat conductance with respect to the charge conductance makes the quantum dot junction potentially the “best thermoelectric” as theorized by Mahan and Sofo.^40^ With increasing tunnel coupling, such that γ > *k*_B_*T*, the transmission function  is broadened, and the energy selectivity is gradually lost, thereby restoring the WF law. A full calculation of *L*/*L*_0_ versus γ/*k*_B_*T* is plotted in the Supporting Information, Figure S6.

We exemplify this gradual recovery of the WF law by studying the heat flow close to the conductance resonances observed at a larger gate voltage *V*_g_. While at *V*_g_ ≈ 2.9 V, a ratio γ/*k*_B_*T*_b_ ≈ 7 placed the device in the intermediate coupling regime, still displaying sizable energy selectivity (Figure 2), at *V*_g_ ≈ 4.1 V the tunnel couplings are about a factor of 2.5 larger (Figure 3a). We therefore expect a gradual transition to a WF-like heat conductance. This is seen in Figure 3a, where we superimpose the experimentally determined *G* and κ_e_ on a vertical scale connecting both quantities via the WF law; that is, κ_e_ = *GT*_b_*L*_0_. At the charge degeneracy points (conduction resonances), we observe that the dimensionless reduced heat conductance *L*/*L*_0_ is now very close to, or barely below, 1. Moving away from the conductance peak, *G* and κ_e_ also superimpose nearly exactly, within noise, as also expected from a scattering transport calculation with a now broader  (line). Observing a sizable deviation from WF requires going beyond linear response (Figure 3b),^41^ where the experimental data and the scattering transport calculation remain nevertheless now much closer to the WF law. The main conclusion we draw here is that for increasing tunnel couplings, the scattering theory still describes the experimental data very accurately and over a large temperature difference range. In the linear response regime (small Δ*T*), the WF law and scattering theory yield convergent predictions.

**Figure 3:**
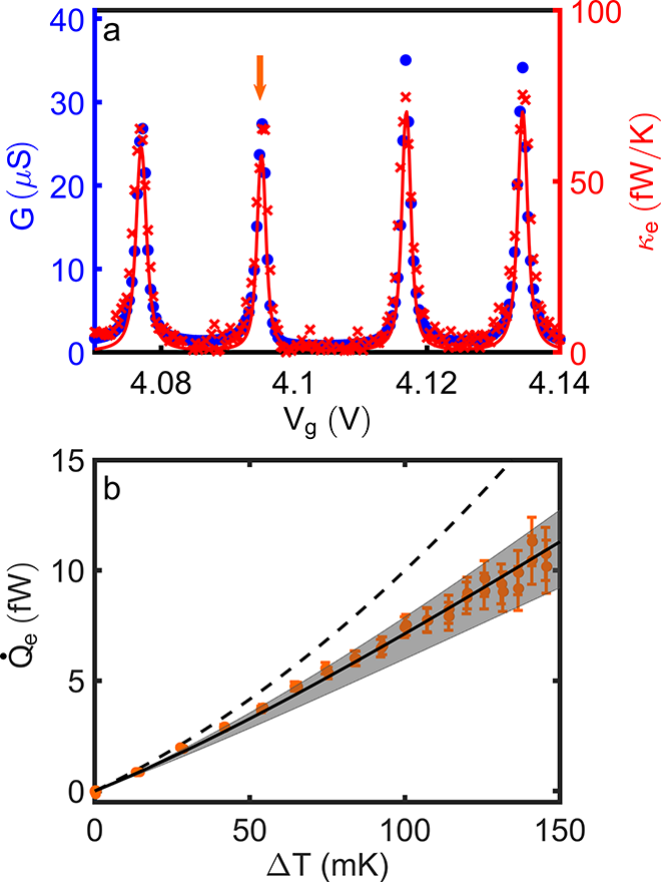
Heat versus charge transport at higher transmissions. (a) Heat (red crosses, right vertical scale) and charge (blue bullets, left vertical scale) conductance resonances at higher transmissions. The ratio of both vertical scales is set to *T*_b_*L*_0_, such that superimposed curves are indicative of the WF law being valid. The red line is the calculated κ_e_ from scattering transport theory. The *L*/*L*_0_ for the four peaks are 0.99, 0.97, 0.87, and 0.90 (±0.05) from left to right. (b) *Q̇*_e_(*T*_e_) curve taken at the conduction resonance at *V*_g_ = 4.095 V (arrow in (a)). The dashed and the full lines are the predictions from the WF law and scattering transport theory, respectively. The gray shaded area indicates the uncertainty of the scattering theory calculation, due to the determination of the gate coupling lever arm. The error bars account for the uncertainty in the experimental determination of *Q̇*_e_.

Moving to yet larger gate voltages (*V*_g_ > 4.5 V) and thus electronic transmissions, the charge conductance no longer vanishes in between conduction resonances, impeding the identification of a clear-cut local reference *Q̇*_e-ph_(*T*_e_). This prevents a quantitative separation of the electronic heat flow through the nanowire from the e-ph contribution.

At the lower gate voltages, we however can estimate the e-ph coupling induced by adding carriers to the nanowire segment below the source. This is precisely captured by the off-resonance *Q̇*(*T*_e_) shown by the green line in Figure 2c, which follows a power law ∝ (*T*_e_^6^–*T*_b_^6^). Interestingly, this leads to an e-ph coupling constant comparable to that of a metal, in spite of the electron density being several orders of magnitude smaller. This finding is consistent with the strong e-ph coupling found in InAs above 1 K^32^ possibly due to piezoelectricity^42^ and/or a lateral-confinement-enhanced peaked density of states.^43^ We observe the e-ph contribution to change linearly with *V*_g_ (see associated plot and analysis in the Supporting Information) implying that the e-ph coupling constant is proportional to the charge carrier density.

In summary, our study reveals a large conjunct evolution in the thermal and charge conductances of an InAs nanowire near pinch off. Around conductance resonances in the quantum dot regime of the nanowire, the heat conductance is significantly lower than expected from the WF law, with κ_e_/(*GTL*_0_) reaching 0.65 in the intermediate coupling regime, in good agreement with a scattering transport calculation. As anticipated by theory,^40^ this establishes experimentally the huge potential of semiconductor nanowires and more generally quantum dot transistors, as promising high-figure-of-merit thermoelectrics. It is interesting to note that while the single-electron transistor (SET) and the quantum-dot junction share extremely similar charge conductance properties in the linear regime, their thermal transport properties show striking differences. At resonance (charge degeneracy), interaction effects are canceled in both types of devices, and the SET thus behaves as a simple metallic heat conductor, whereas the quantum dot junction displays a heat conductance suppression below the WF law. Off resonance, however, Coulomb blockade leads the SET to behave like a high-pass filter in energy (as opposed to the single quantum level, which can be viewed as a band-pass filter), which leads to a heat conductance exceeding the WF law.^11,12^ A fascinating open question resides in the role played by electron interactions and correlations in quantum dots,^7,19^ which are also expected to lead to marked deviations from the here-employed scattering transport picture, away from the conduction resonances.

## Supporting Information Available

Additional information about the sample fabrication, the electron thermometry, the scattering transport calculations, the determination of the quantum dot parameters, and the analysis of the heat balance (PDF)

## Notes

All data described here will be made publicly available on Zenodo.
