# Supplementary material for: Quantum Confinement Suppressing Electronic Heat Flow below the Wiedemann–Franz Law
Source: Nano Lett. 2022 Jan 14;22(2):630–5. doi: 10.1021/acs.nanolett.1c03437 (PMC8802316; doi:10.1021/acs.nanolett.1c03437)
Supplement: Supplementary file 1 — nl1c03437_si_001.pdf [file nl1c03437_si_001.pdf]

Supplemental material for the article entitled "Quantum confinement suppressing electronic heat flow below the Wiedemann-Franz law"

Danial Majidi,[†] Martin Josefsson,[‡] Mukesh Kumar,[‡] Martin Leijnse,[‡] Lars Samuelson,[‡] Hervé Courtois,[†] Clemens B. Winkelmann,[*,†] and Ville F. Maisi[*,‡]

† *Univ. Grenoble Alpes, CNRS, Grenoble INP, Institut Néel, 25 rue des Martyrs, Grenoble, France*

‡ *NanoLund and Solid State Physics, Lund University, Box 118, 22100 Lund, Sweden*

E-mail: clemens.winkelmann@neel.cnrs.fr; ville.maisi@ftf.lth.se

Abstract

We provide here supplemental material and information concerning the sample fabrication process, details of the electronic thermometry and heating, a description of the Landauer-Büttiker transport calculations, extended charge transport data of the quantum dot junction, and details of the thermal balance analysis.

Sample Fabrication

Our device consists of a InAs nanowire with a 70 nm diameter that was grown by chemical beam epitaxy seeded by a gold catalyst particle[1]. The device is fabricated with two rounds of electron beam lithography (EBL) and subsequent hydrofluoric acid (HF) passivation and metal depositions. The substrate is a p-doped (resistivity $1 - 30\Omega cm$), single-side polished

2" Si wafer with 200 nm oxide allowing the underlying Si substrate to be used as a global back gate. In the first lithography round, a bulky drain (shown in green in Fig. S1) and part of the source (visible as a circle on the right hand side of the nanowire) are patterned. Subsequently, HF passivation (5 s in BOE 1:10 followed with fast rinsing in deionized water and immediate loading to the metal evaporator) is performed before contacts are metallized thermally as a stack of Ni (30 nm)/ Au (60 nm). The Ni layer helps with adhesion to the SiO_2 substrate and formation of an electrical contact to the nanowire.

Following a standard lift-off, a suspended P(MMA-MAA) copolymer based mask was spin-coated for the next EBL step where all the NIS junctions and the source island are defined as shown in Fig. S1 in blue and red color. After e-beam lithography, the mask is again loaded into an evaporator equipped with a tiltable sample holder. This allows fabricating both the normal-metal island (red) and the superconducting leads (blue) using the same mask and in a single vacuum cycle. First, a 35 nm thick film of Al is deposited at $+11^{\circ}$ with respect to the evaporation source. It is indicated in Fig. S1 in blue color. To form the AlOx tunnel barriers for NIS probe tunnel junctions, the deposited Al layer is subjected to in-situ static oxidation immediately after the deposition is completed. This was accomplished by venting the chamber at air followed by an immediate pumping of the system. To complete the fabrication, a 60 nm thick Cu film was evaporated with the sample now tilted to -11° in the opposite direction compared to the Al deposition. This upward-shifted copy of the mask pattern forms the source island (colored in red in Fig. S1). The purpose of this Cu layer is to form the main part of the source electrode, connecting to the small source lead which was deposited in the first step. As a result of the two-angle evaporation through the same mask, two projections of the complete mask pattern will be formed on the substrate. The irrelevant, partially overlapping shadow copies of the various structures, evident in Fig. S1 are shown uncolored.

The leftmost Al electrode in Fig. S1 overlaps with the circular part of the source electrode made in the first lithography round. It therefore connects with a transparent contact to the

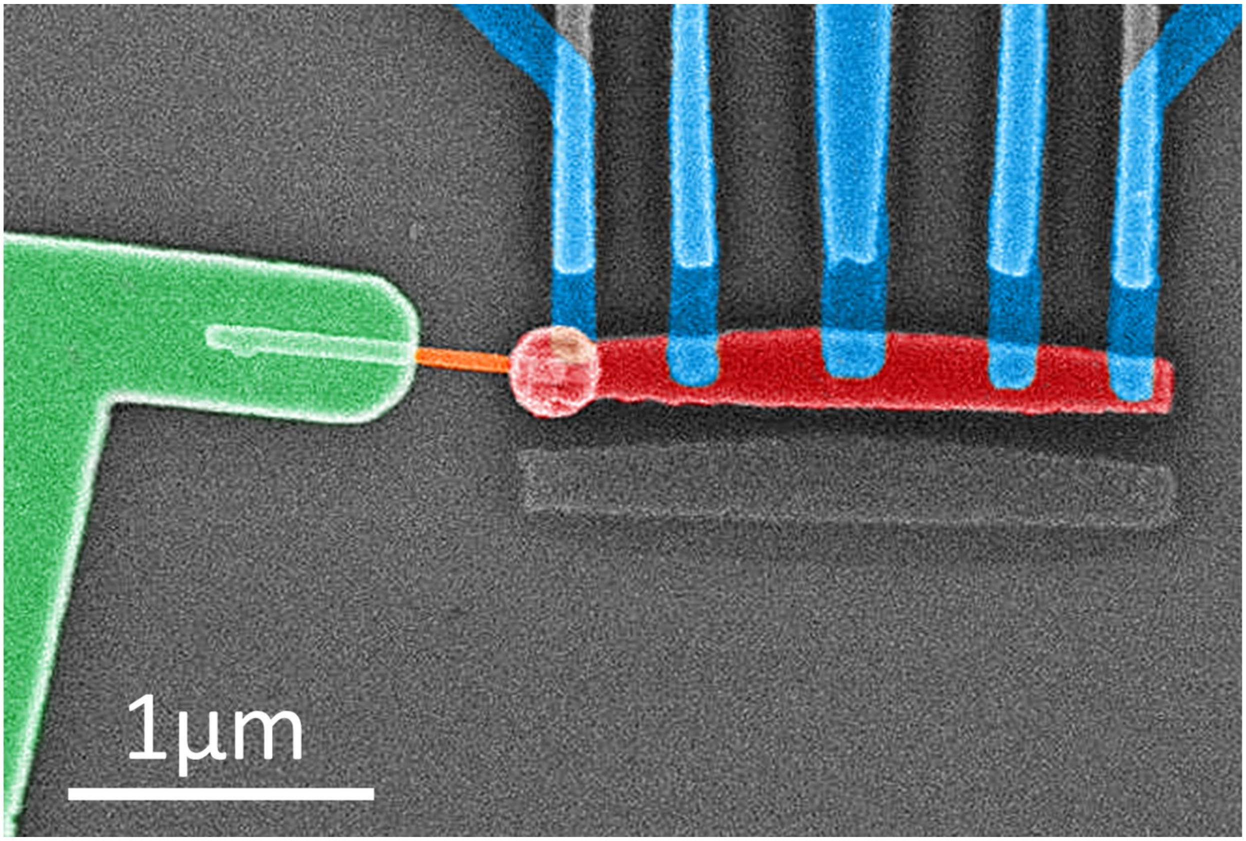

Figure S1: False-color scanning electron micrograph of the InAs nanowire device realized with two steps fabrication and shadow-evaporated Al-proximity junctions.

source island without the oxidation, whereas the others connect via the oxide tunnel barrier through the Cu part of the source and hence display a large tunnel resistance. The leftmost lead allows for probing the charge transport of the nanowire. We do not observe any sign of a superconducting proximity effect on the nanowire caused by this electrode. This is most likely due to the fact that the aluminum contact is much thinner (35 nm) than the nickel/gold island (90 nm) connecting the nanowire.

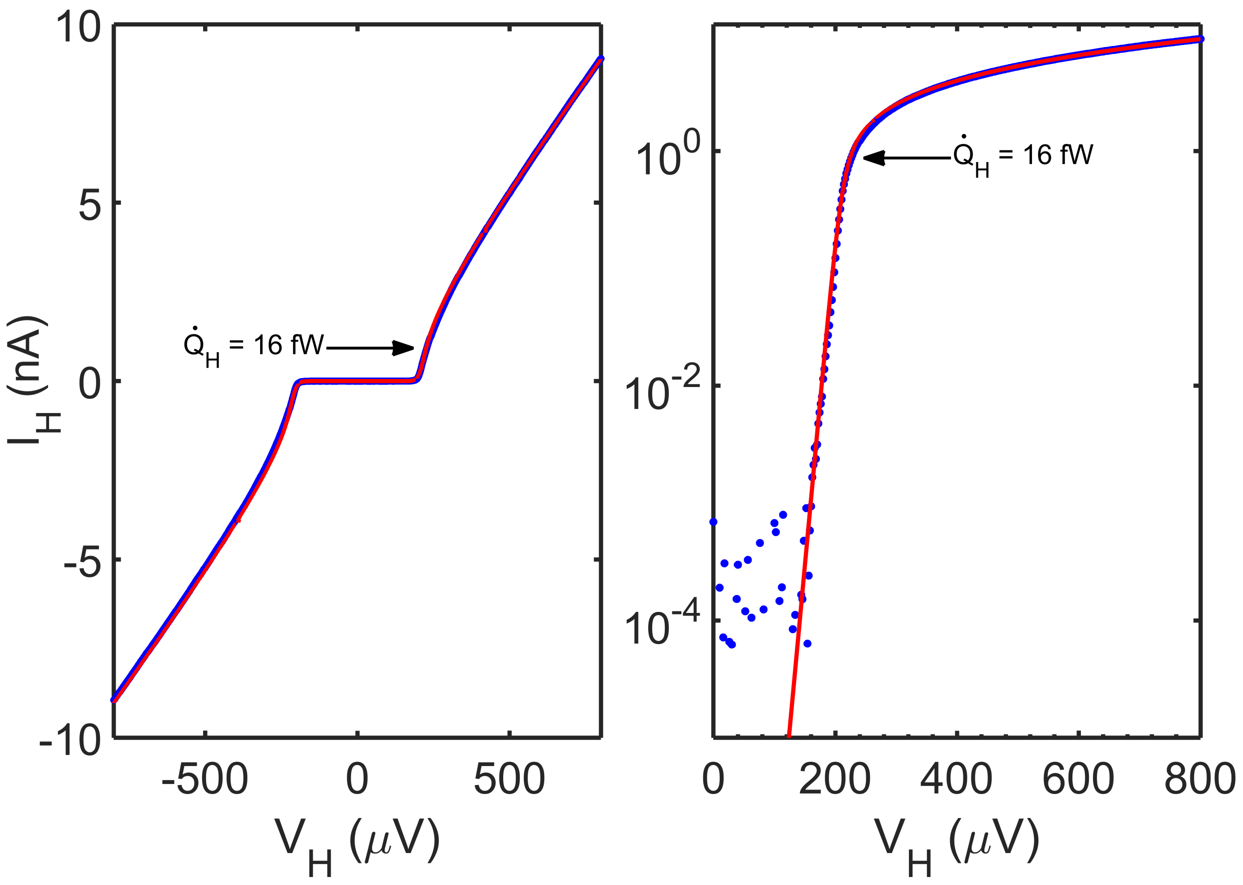

Figure S2: Current–voltage curve of the heater NIS junction using linear (left) and logarithmic (right) scale. Fit to Eq. (S1) is shown as red lines: $\Delta = 209\mu ev$, $R_T = 85.6$ kΩ and T_b= 100 mK. A typical heater current level of I_H= 0.9 nA resulting in $\dot{Q}_H = 16$ fW and $\Delta T \approx 50$ mK (see Figs. 1 and 2 of the main article) is indicated with a black arrow.

NIS **THERMOMETER AND HEATER CHARACTERIZATION**

In this section, we describe the functioning of the NIS junction as heaters and thermometers, and how their parameter values were extracted. The latter include the normal state tunnel resistance R_T, and the low-temperature superconducting energy gap Δ. The I-V

characteristics and heat current through a single NIS junction reads as[2], respectively

$$I = \frac{1}{2eR_T} \int_{-\infty}^{+\infty} n_s(E, \Delta) \times [f_N(E - eV) - f_N(E + eV)] \, dE \tag{S1}$$

and

$$\dot{Q}_H(E) = \frac{1}{e^2 R_T} \int_{-\infty}^{+\infty} (E - eV) \, n_s(E, \Delta) \times [f_N(E - eV) - f_S(E)] \, dE \tag{S2}$$

where $n_S(E)$ is the normalized BCS density of states with an energy gap of Δ, and $f_S(E)$ and $f_N(E)$ are the quasi-particle occupation factors for the superconductor and normal metal. It is worth mentioning that the charge current depends on the electronic temperature of the normal metal but not on the temperature of the superconductor for temperatures well below Δ/k_B. Despite the apparent simplicity of the above expressions, they produce quantitatively correct predictions in most experimentally interesting cases. The low-temperature experimental characteristic of the heater NIS junction of our device at $T_b = 100$ mK is shown in Fig. S2, both on linear and logarithmic scale, together with the theoretical $I - V$ characteristic using Eq. (S1). We see that the theory line catches all features except at the lowest currents where the noise of the current preamplifier contributes to the scatter of the data points.

The heating of the source island was made by applying a voltage V_H as shown in Fig. 1a of the main article. The ensuing power $\dot{Q}_H$ is given by Eq. (S2). A peculiar property of the NIS junctions is that for bias voltages slightly less than the gap voltage Δ/e, one can find a region where $\dot{Q}_H$ is negative, i. e., the normal electrode is cooled. This Peltier effect has been extensively studied[2]. Due to uncertainties in the precise determination of the power at sub-gap voltages, we however have not included data for negative $\dot{Q}_H$ in our heat balance analysis but rather focus on the heating side.

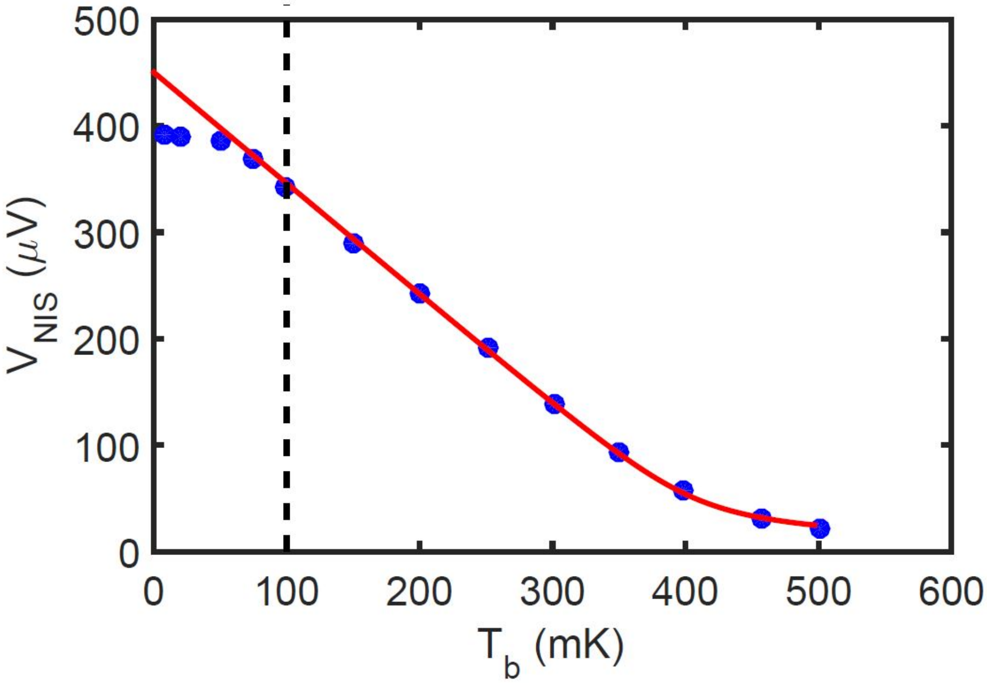

Figure S3: Measured voltage across the thermometer junctions with a floating current of $I_{\mathrm{NIS}} = 5$ pA as a function of the bath temperature T_b. The dashed line presents the bath temperature $T_b = 100$ mK used in the heat flow measurements. The red line shows the expected response of Eq. (S1) with $\Delta = 209$ μeV and $R_{T1} = R_{T2} \sim 96.2$ $k\Omega$ obtained from a similar fit as in Fig. S2.

Thermometry is performed by using two NIS junctions in series (SINIS). We bias the pair of the NIS junctions with a constant current of $I_{\mathrm{NIS}} = 5$ pA and measure the voltage drop V_{NIS} across the junctions to determine the electron temperature of the source island[2]. The thermometer is calibrated by varying the bath temperature T_b of the cryostat. The calibration is done at equilibrium without heating the source island, so that the electronic temperature of the source follows the cryostat temperature (equal to the substrate phonon temperature) and results in the response presented in Fig. S3. The voltage V_{NIS} changes as a result of thermal excitations on the normal metal lowering the voltage from the low temperature threshold value corresponding to approximately the superconductor gap $\Delta/e \approx 200$ μV per junction. At low $T_b < 50$ mK, we indeed see the saturation at $V_{\mathrm{NIS}} \approx 400$ μV. All our measurements are performed at $T_e \geq 100$ mK, making sure that the thermometer operates well above the low temperature saturation.

Analysis of transport through a single quantum dot orbital

As evidenced by the conductance peaks (see figures in the main paper) and by the stability diagrams (Fig. S5) a quantum dot forms in the nanowire below $V_g = 4.5$ V. From the stability diagrams we find charging energies $E_c \approx 1.5 - 2$ meV. Additional sequential tunneling and cotunneling resonances show that, in contrast with metallic islands, there is also a substantial energy splitting between the quantized quantum dot orbitals, $\Delta\varepsilon \gg \gamma, kT$.

The energy scales of our device in the quantum dot regime, with $E_c \gg \gamma > k_B T$, unfortunately prevents the use of theoretical approaches based on perturbation theory in γ, and make theories that include E_c on an approximate footing unreliable. Our approach is instead to model the QD with a non-interacting model close to the charge-degeneracy points (Coulomb peaks). In this case the currents through a *single* quantum dot orbital can be calculated using Landauer-Büttiker transport theory as[3,4],

$$I = \frac{2e}{h} \int_{-\infty}^{\infty} \mathcal{T}(E) \cdot \Delta f \; dE, \tag{S3}$$

$$\dot{Q}_e = \frac{2}{h} \int_{-\infty}^{\infty} (E - \mu_s) \cdot \mathcal{T}(E) \cdot \Delta f \; dE, \tag{S4}$$

where

$$\Delta f = f_s - f_d, \quad f_n = \left(\exp\left(\frac{E - \mu_n}{k_b T_n} \right) + 1 \right)^{-1}, \tag{S5}$$

and

$$\mathcal{T}(E) = \frac{4\gamma_s \gamma_d}{\gamma^2} \frac{\left(\frac{\gamma}{2}\right)^2}{(E - (\varepsilon - eV_g))^2 + \left(\frac{\gamma}{2}\right)^2}, \tag{S6}$$

with $\gamma = \gamma_s + \gamma_d$. In Eq. (S6) we assumed that $\gamma_{s,d}$ are energy-independent. Note that we above analysis allows finding the values of the pair (γ_s, γ_d), but does not allow assigning which one is which. We therefore list the tunnel couplings as $\gamma_{1,2}$ from hereon, without specifying which one is γ_s and γ_d, respectively.

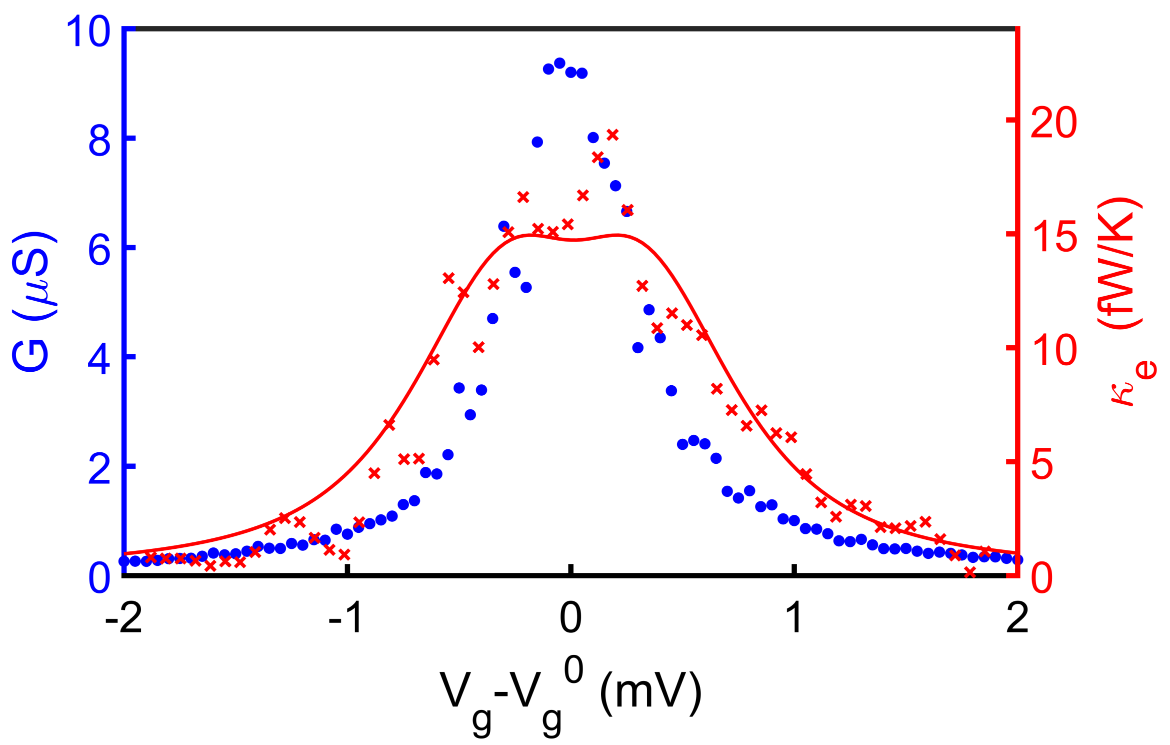

Figure S4: Heat (red crosses,right vertical scale) and charge (blue bullets, left vertical scale) conductance resonances at higher transmissions. The ratio of both vertical scales is set to $T_b L_0$, such that super imposed curves are indicative of the WF law being valid. The red line is the calculated κ_e from scattering transport theory.

The relevant physical quantities for this study are the linear electrical and thermal conductances, $G = \frac{\partial I}{\partial V}\Big|_{\Delta T=0}$ and $\kappa_e = \frac{\partial \dot{Q}_e}{\partial(\Delta T)}\Big|_{I=0}$, which are obtained by differentiating Eqs. (S3, S4). The experimental data of $G(V_g)$ and $\kappa_e(V_g)$ around the resonance near $V_g = 2.938$ V, along with the full non-interacting calculations are shown in Fig. S4.

Quantum dot characterisation

In the quantum dot regime (at small V_g), the conductance map provides all information needed to determine the parameters of the quantum dot. Fig. S5 displays the measured differential conductance maps (obtained by numerical differentiation) as a function of both the bias and gate voltages V_b and V_g, respectively, around the operation points of Figs. 2

and 3 of the main article. Coulomb diamonds can be seen clearly and the charging energy of $E_c \sim 1.5 - 2$ meV is estimated from extrapolating the bias level to the top of a diamond.

The positive slope of the Coulomb diamonds in Fig. S5 is given by $\beta = \frac{C_g}{C_d + C_g}$ and the negative one is given by $\beta' = \frac{C_g}{C_s}$. Here the C_s, C_d, C_g are the capacitances between the dot and source, drain and the gate respectively. The total capacitance of the dot to the outside world is the sum of all capacitances as $C_\Sigma = C_s + C_d + C_g$. An important parameter in the calculations is the lever arm defined by the ratio $\alpha = \frac{C_g}{C_\Sigma}$ as it translates changes in gate voltage to energy changes for electrons on the quantum dot, $\Delta\varepsilon = -e\alpha\Delta V_g$. From the conductance maps close to $V_g \sim 4.1$ V and $V_g \sim 3$ V we extract a local lever arm α_m from the slopes of conductance lines. We observe that the level arm value varies slightly with the gate voltage, see Tab. S1. Therefore, to account for uncertainties in the determination of gate coupling, we also performed the full theoretical analysis at $\alpha_m \pm 0.01$ for $V_g \sim 3$ V and $\alpha_m \pm 0.02$ for $V_g \sim 4.1$ V.

Table S1: Extracted lower (α_l) and upper (α_u) bounds of gate couplings for the Coulomb peaks, and their mean value (α_m) at the resonances considered in the main article.

$V_g = 2.933$ V	$V_g = 4.095$ V	$V_g = 4.117$ V
$\alpha_l = 0.095$	$\alpha_l = 0.06$	$\alpha_l = 0.06$
$\alpha_m = 0.105$	$\alpha_m = 0.08$	$\alpha_m = 0.08$
$\alpha_u = 0.115$	$\alpha_u = 0.1$	$\alpha_u = 0.1$

Next we extract a pair of tunnel couplings (γ_1, γ_2) for each Coulomb peak by fitting the calculated zero-bias conductance (obtained using Eqs. S3 and S6) as a function of the gate voltage to the measured counterpart. The parameter values for the two tunnel couplings are obtained uniquely from the height $\frac{\gamma_1\gamma_2}{\gamma_1+\gamma_2}$ and the width $\gamma_1 + \gamma_2$ of the transmission function, which corresponds roughly to the height and width of the Coulomb peak. Since we have already determined the appropriate α this fitting process involves no additional fitting parameters. When performing these fits, we restrict the G data to only cover a single Coulomb peak and use $T_b = T_s = T_d = 100$ mK, which was the temperature of the device during the measurement of G. The resulting best fits obtained using α_m are shown in Fig. 2

of the main paper for $V_g \sim 3$ V and in Fig. S6 for the resonances at $V_g \sim 4.1$ V corresponding to the data presented in Fig. 3 of the main paper. The extracted values are shown in Tab. S2.

Around 4.1 V (Fig. 3 of the main article), the total transmission function $\mathcal{T}(E)$ of the device is taken to be the sum of the four individual Lorentzian transmission functions for each Coulomb peak, centered around the V_g values listed in Tab. S2.

Table S2: Extracted tunnel couplings for the Coulomb peaks in two regimes $V_g \sim 2.938$ V, and $V_g \sim 4.1$ V.

V_g (V)	α	γ_1 (μeV)	γ_2 (μeV)
2.938	$\alpha_m = 0.105$	55.5	2.1
4.077	$\alpha_m = 0.08$	137.5	15.5
4.095	$\alpha_m = 0.08$	105.9	12.4
4.117	$\alpha_m = 0.08$	104.8	15.9
4.13	$\alpha_m = 0.08$	122.9	17.5

In addition to modelling the device at the operating conditions of the experiment, the theory also allows us to investigate how the Wiedemann-Franz law violations scale with system parameters. Focusing on the resonant condition, i.e. gating the device to the middle of a conductance peak, we calculate how L/L_0 scales with the width $(\gamma_1+\gamma_2)$ and amplitude $(\frac{\gamma_1\gamma_2}{\gamma_1+\gamma_2})$ of $\mathcal{T}(E)$. The result is shown in Fig. S7, where the theory predictions for the five resonances analyzed in this work are also highlighted. From the figure it is evident that there are two paths for decreasing the L/L_0 ratio: lowering $\gamma_1 + \gamma_2$ or increasing k_BT. However, lowering the tunnel couplings is non-trivial in our device as the quantum dot forms spontaneously at low carrier concentrations and there is little experimental control over the coupling strength. In addition, an effect of overall lower values of γ_1 and γ_2 is a reduced heat flow, which can be hard to detect experimentally since the signal is more easily swallowed by the noise-floor. The other approach, to increase k_BT, also has its limitations as the NIS thermometer requires the Al leads to be well below the critical temperature of the superconductor and the e-ph coupling of the source island to be small[2]. One can thus

conclude that the present device is very well suited for studying violations of the Wiedemann-Franz law due to quantum confinement given the constraints of the state-of-art technologies used in the study.

Determination of the heat flows, analysis of the electron-phonon couplings

The relation $\dot{Q}_H(T_e, V_g = 0)$ between the applied heating power and the source island electronic temperature at $V_g = 0$ is shown in Fig. S8. The good quantitative agreement with an electron-phonon type thermal law[2] shows that electron-phonon coupling must be the dominant thermal leakage channel out of the source island, in the absence of electronic heat conduction through the nanowire. The red curve is a fit with $\dot{Q}_H = \Sigma\Omega({T_e}^5 - {T_b}^5)$. By using the geometrically estimated total volume $\Omega = 4.26 \pm 0.2 \times 10^{-20}$ m^3 of the source island, we obtain the fitted value $\Sigma = 2.5 \pm 0.1 \times 10^9$ Wm^{-3}K^{-5} of the average electron-phonon coupling coefficient in the metallic source island, in good agreement with the expected coupling coefficients of Cu and Au , that is, $\Sigma \approx 2.0 \times 10^9$ Wm^{-3}K^{-5} and 2.4×10^9 Wm^{-3}K^{-5}, respectively[2].

At a given resonance peak, the electronic heat conductance is experimentally determined by subtracting a local reference of heat $\dot{Q}$ measured close to the resonance at a point where the electronic contribution is negligible. We have checked that, owing to the narrowness of the conduction resonances, taking the reference on a single side, or as an average over left and right, yields indistinguishable results, as seen in Fig. S10. Fig. S9 shows that the heat flow is constant within noise at low electrical conductance G far enough away from the main peak although G changes by one order of magnitude. Only close to the resonance peak, we observe an extra contribution identified as the electronic part. On both sides of the peak, $\dot{Q}$ has the same background level when G is small enough. It is also worth noting that the variation of the background contribution is vanishingly small for $\Delta T < 40$ mK as seen from

Fig. 2c of the main article. For small ΔT, the electronic contribution dominates.

In order to understand this background contribution, we have analysed the $\dot{Q}(T_e, V_g)$ curves in the entire non-conducting regime of the nanowire. For this purpose, we have focused on the regime between $V_g = 0 \ldots 4.5$ V, excluding conduction resonances, that is, data sets at values of V_g at which $G > 0.5$ μS. The background part of the heat flow increases steeply at $\Delta T \gtrsim T_b$ and is related to electron-phonon coupling in the nanowire following a $\propto (T_e^6 - T_b^6)$ law provided by far the best agreement. Because on the microscopic level the e-ph coupling can be quite different in InAs and the metallic island, it is not surprising that we observe a different exponent for the e-ph coupling of both systems[2].

The prefactor β is plotted in Fig. S10. As expected, β increases smoothly with V_g, supporting the hypothesis of a dependence on the carrier concentration in a segment of the nanowire not belonging to the quantum dot. This could be for example the portion of the nanowire underneath the source island, of volume $V = 7.7 \times 10^{-22}$ m^3. Making this assumption, the e-ph heat conductance per unit volume in the metallic source is on the same order of magnitude as that of the nanowire. Note that our method is probably underestimating β by a constant shift, since we assumed its value to be 0 at $V_g = 0$ V.

References

(1) Björk, M. T.; Thelander, C.; Hansen, A. E.; Jensen, L. E.; Larsson, M. W.; Wallenberg, L. R.; Samuelson, L. Few-electron quantum dots in nanowires. *Nano Lett.* **2004**, *4*, 1621–1625.

(2) Giazotto, F.; Heikkilä, T. T.; Luukanen, A.; Savin, A. M.; Pekola, J. P. Opportunities for mesoscopics in thermometry and refrigeration: Physics and applications. *Rev. Mod. Phys.* **2006**, *78*, 217.

(3) Sivan, U.; Imry, Y. Multichannel Landauer formula for thermoelectric transport with application to thermopower near the mobility edge. *Phys. Rev. B* **1986**, *33*, 551.

(4) Davies, J. H. *The physics of low-dimensional semiconductors: an introduction*; Cambridge University Press, Cambridge, 1998.

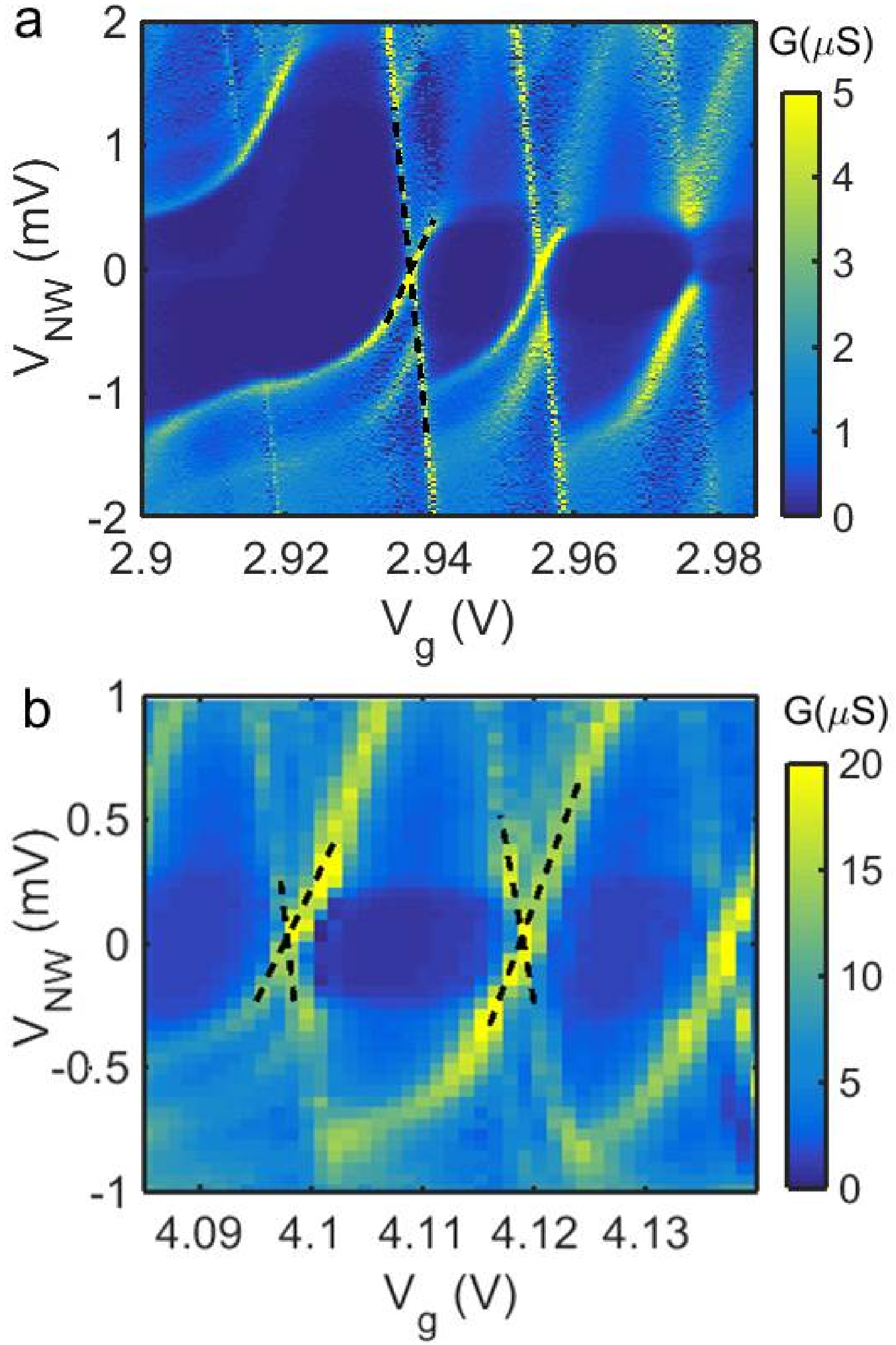

Figure S5: Differential conductance maps near $V_g \sim 2.9$ V (raw data) (a) and $V_g \sim 4.1$ V (interpolated data) (b). The bath temperature T_b is 100 mK.

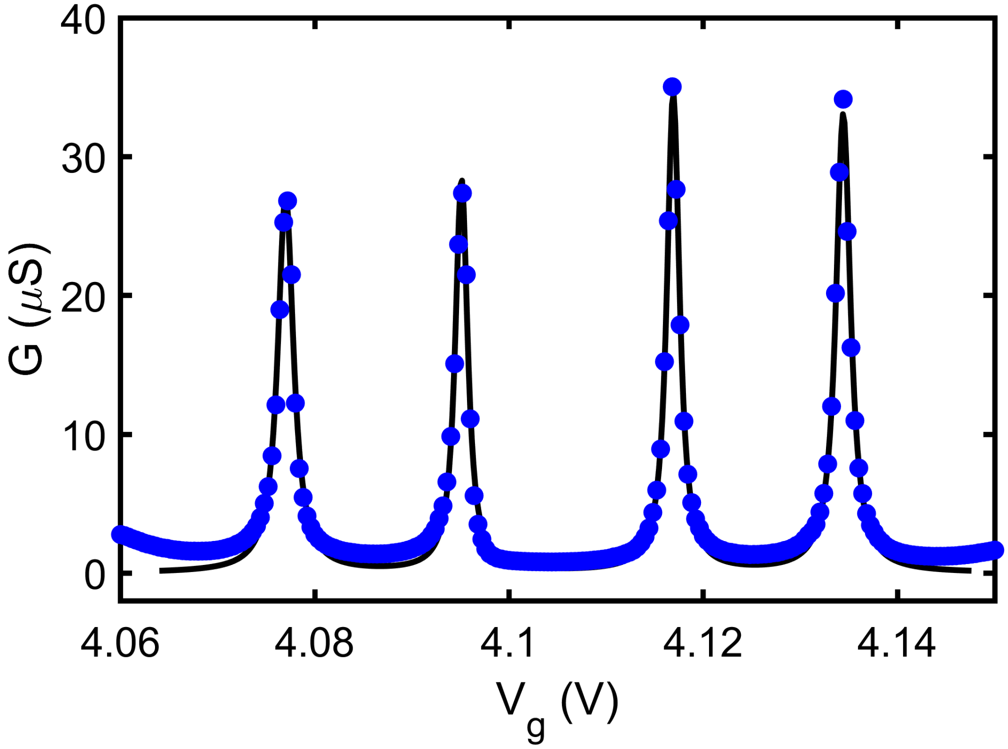

Figure S6: Measured (blue markers) and calculated (solid line) charge conductance of the device around the charge degeneracy points close to 4.1 V. The full transmission function used for the theory prediction is obtained by combining one $\mathcal{T}(E)$ for each peak, determined by fitting the calculated G to the measured data in the vicinity of a single peak.

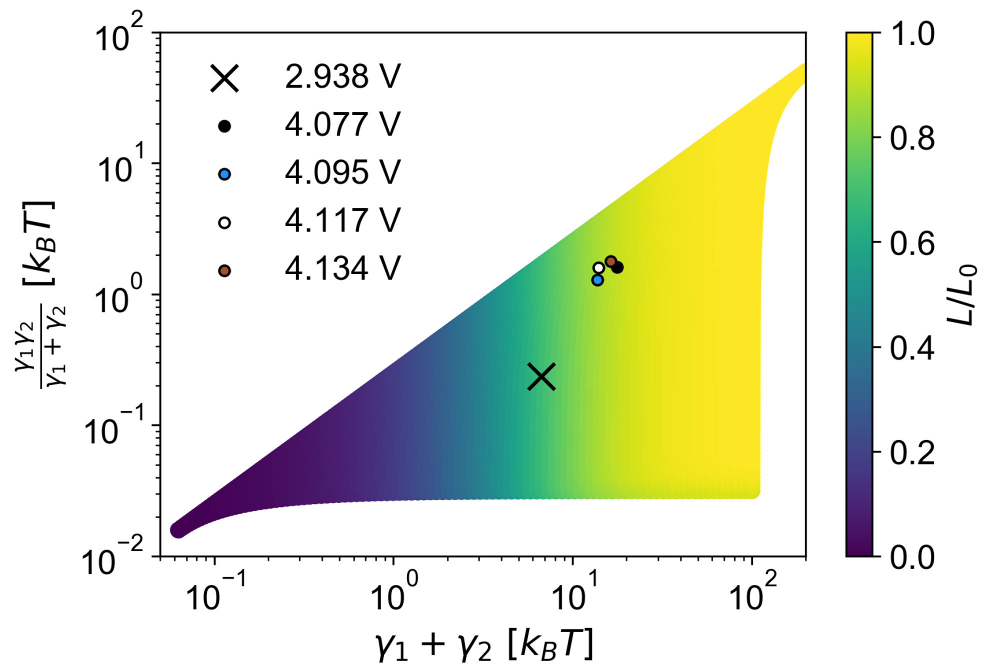

Figure S7: Calculated L/L_0 on resonance as a function of the width $(\gamma_1 + \gamma_2)$ and amplitude $(\frac{\gamma_1\gamma_2}{\gamma_1+\gamma_2})$ of $\mathcal{T}(\mathrm{E})$. The markers show the theoretical predictions for the different resonances studied in the main manuscript.

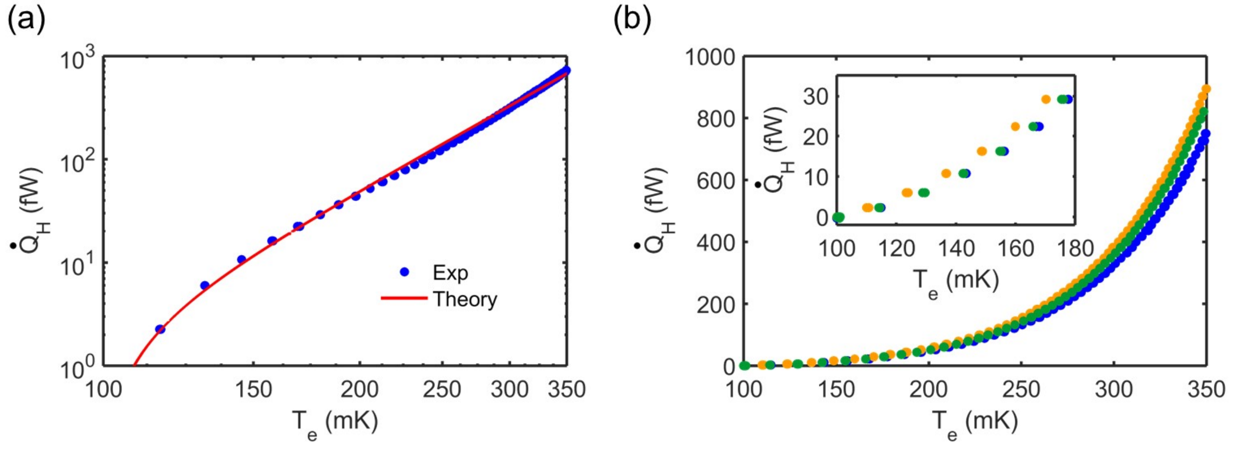

Figure S8: Raw data of the heating power $\dot{Q}_H$ applied to the source island versus the electron temperature T_e in the source island, at $T_b = 100$ mK. In (a) $\dot{Q}_H$ is shown at $V_g = 0$ V and on a log scale, together with a fit using the $\dot{Q}_H = \Sigma\Omega({T_e}^5 - {T_b}^5)$ heat balance law (see text). In (b) $\dot{Q}_H$ versus T_e is shown on a linear scale, at $V_g = 0$ V (blue), $V_g = 2.90$ V (green, near but slightly off the first conduction resonance) and $V_g = 2.938$ V (orange, on resonance). The inset is a zoom on the low temperature regime.

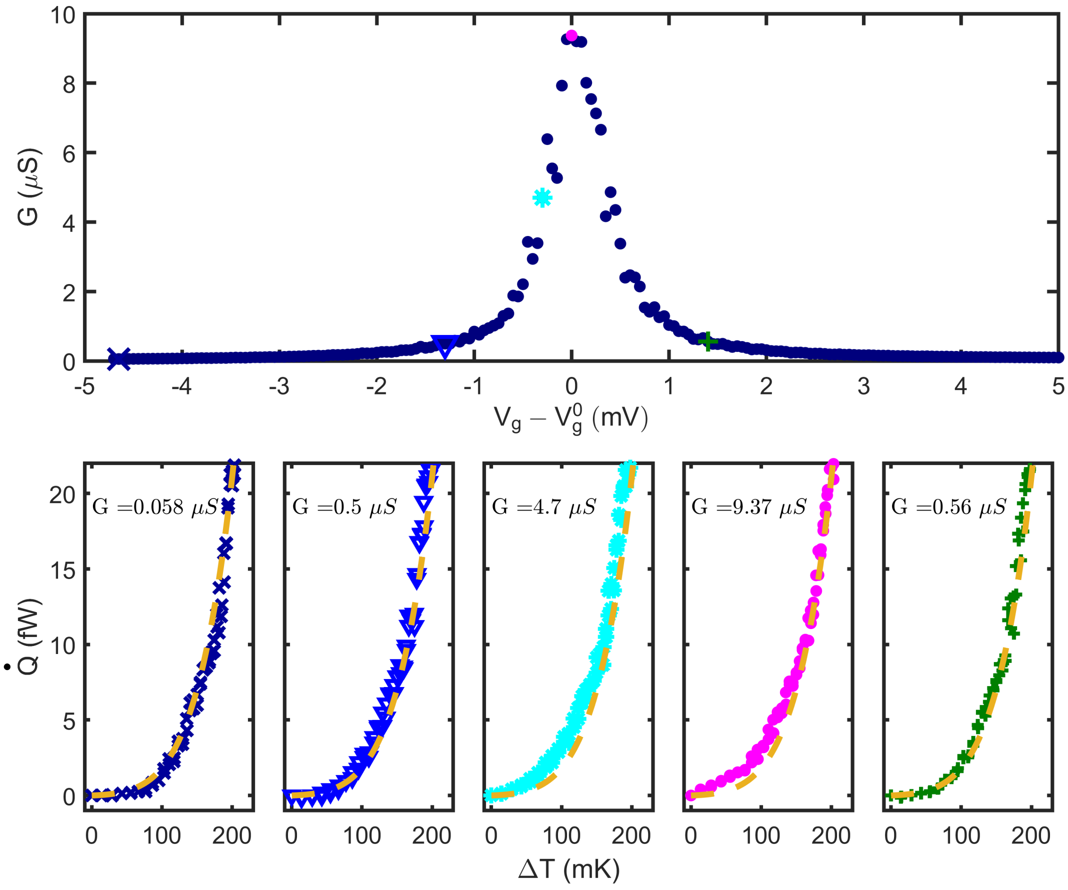

Figure S9: Top : Charge conductance G peak around the resonance at ${V_g}^0 = 2.938$ V. Bottom : heat flow $\dot{Q}$ as a function of the temperature difference at several values of the gate potential indicated by color symbols in the bottom panel. The dashed line is the best $\propto (T_e^6 - T_b^6)$ fit obtained from the data in the leftmost sub-panel, and displayed identically in all sub-panels for reference.

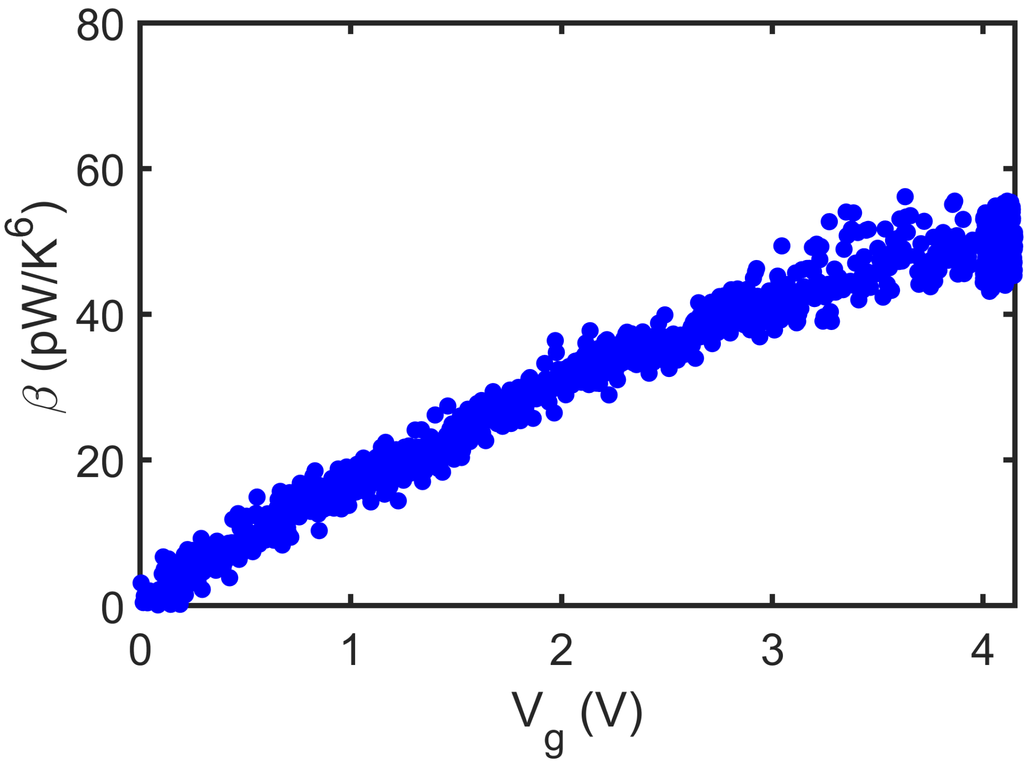

Figure S10: Gate dependence of the electron-phonon coupling: β is extracted by fitting $\dot{Q}(T_e, V_g)$ at each V_g (excluding conduction resonances) with a $\beta(T_e^6 - T_b^6)$ power law.
